# The International Scientific Association for Probiotics and Prebiotics (ISAPP) consensus statement on the definition and scope of synbiotics

**DOI:** 10.1038/s41575-020-0344-2

**Published:** 2020-08-21

**Authors:** Kelly S. Swanson, Glenn R. Gibson, Robert Hutkins, Raylene A. Reimer, Gregor Reid, Kristin Verbeke, Karen P. Scott, Hannah D. Holscher, Meghan B. Azad, Nathalie M. Delzenne, Mary Ellen Sanders

**Affiliations:** 1 Department of Animal Sciences, University of Illinois at Urbana-Champaign, Urbana, IL USA; 2 Department of Food and Nutritional Sciences, The University of Reading, Reading, UK; 3 Department of Food Science and Technology, University of Nebraska – Lincoln, Lincoln, NE USA; 4 Faculty of Kinesiology and Department of Biochemistry and Molecular Biology, Cumming School of Medicine, University of Calgary, Calgary, Alberta Canada; 5 Lawson Health Research Institute, University of Western Ontario, London, Ontario Canada; 6 Translational Research in Gastrointestinal Disorders, KU Leuven, Targid, Leuven, Belgium; 7 Leuven Food Science and Nutrition Research Centre, Leuven, Belgium; 8 Rowett Institute, University of Aberdeen, Aberdeen, UK; 9 Department of Food Science and Human Nutrition, University of Illinois at Urbana-Champaign, Urbana, IL USA; 10 Children’s Hospital Research Institute of Manitoba, Department of Pediatrics and Child Health, University of Manitoba, Winnipeg, Manitoba Canada; 11 Metabolism and Nutrition Research Group, Louvain Drug Research Institute, Université catholique de Louvain, Brussels, Belgium; 12 International Scientific Association for Probiotics and Prebiotics, Centennial, CO USA

**Keywords:** Nutritional supplements, Microbiota, Nutrition therapy

## Abstract

In May 2019, the International Scientific Association for Probiotics and Prebiotics (ISAPP) convened a panel of nutritionists, physiologists and microbiologists to review the definition and scope of synbiotics. The panel updated the definition of a synbiotic to “a mixture comprising live microorganisms and substrate(s) selectively utilized by host microorganisms that confers a health benefit on the host”. The panel concluded that defining synbiotics as simply a mixture of probiotics and prebiotics could suppress the innovation of synbiotics that are designed to function cooperatively. Requiring that each component must meet the evidence and dose requirements for probiotics and prebiotics individually could also present an obstacle. Rather, the panel clarified that a complementary synbiotic, which has not been designed so that its component parts function cooperatively, must be composed of a probiotic plus a prebiotic, whereas a synergistic synbiotic does not need to be so. A synergistic synbiotic is a synbiotic for which the substrate is designed to be selectively utilized by the co-administered microorganisms. This Consensus Statement further explores the levels of evidence (existing and required), safety, effects upon targets and implications for stakeholders of the synbiotic concept.

## Abstract (web summary)

Gut microbiota can be manipulated to benefit host health, including the use of probiotics, prebiotics and synbiotics. This Consensus Statement outlines the definition and scope of the term ‘synbiotics’ as determined by an expert panel convened by the International Scientific Association for Probiotics and Prebiotics in May 2019.

## Introduction

Notable properties of the gut microbiota include its functionality and resilience^1^. A stable gut community protects the host against invading microorganisms and helps maintain homeostasis, including immune regulation. Nonetheless, disruptions occur owing to dietary shifts, antibiotic use, age or infection, leading to a gut microbiota that can contribute to a range of inflammatory, pathogenic and metabolic conditions such as inflammatory bowel diseases, colorectal cancer, metabolic syndrome and atopy^2^. Several strategies have been proposed to modulate the composition and/or function of the gut microbiota, including faecal microbiota transplants, the application of probiotics and other live microorganisms, and the use of non-digestible dietary substrates such as prebiotics^3,4^.

When the synbiotic concept was first described 25 years ago, the notion that selectively fermentable non-digestible food ingredients (prebiotics) could be combined with probiotics was envisioned^5^. Thus, synbiotics were loosely defined as mixtures of “probiotics and prebiotics that beneficially affect the host”^5^. The term itself was formed from the Greek prefix ‘syn’, meaning ‘together’ and the suffix ‘biotic’, meaning ‘pertaining to life’. Despite the availability of similarly worded definitions, confusion exists among stakeholders, including scientists, about what constitutes a synbiotic^6–9^. A general misunderstanding might have been, in part, because the original definition itself — that is, “mixtures of probiotics and prebiotics that beneficially affect the host by improving the survival and implantation of live microbial dietary supplements in the gastrointestinal tract, by selectively stimulating the growth and/or by activating the metabolism of one or a limited number of health-promoting bacteria, thus improving host welfare” — was too wordy and lacked precision^5^. In addition, the expansion of the entire ‘–biotics’ category, including terms such as postbiotic^10^ and pharmabiotic^11^, almost certainly further contributes to confusion. To provide clarity and guidance regarding appropriate use of the term ‘synbiotic’, in May 2019, the International Scientific Association for Probiotics and Prebiotics (ISAPP) convened an expert panel of academic scientists to address the current status of synbiotics, including its definition. The outcomes of that meeting and subsequent discussions comprise this Consensus Statement. A summary of key conclusions is shown in Box 1. Herein, we consider applicable efficacy and mechanistic evidence on combination probiotic plus prebiotic products, we recommend the research needed to establish a ‘synbiotic’ formulation, discuss the safety considerations, and reflect on implications for stakeholders of the synbiotic concept.

Box 1 Main conclusions of the consensus panel regarding synbiotics

- The definition of synbiotic has been updated to “a mixture comprising live microorganisms and substrate(s) selectively utilized by host microorganisms that confers a health benefit on the host”.
- Within this definition, ‘host’ microorganisms comprise both autochthonous (resident or colonizing the host) and allochthonous (externally applied, such as probiotics) microorganisms, either of which can be targets for the substrate contained in the synbiotic.
- Two subsets of synbiotics were defined: complementary and synergistic. A ‘synergistic synbiotic’ is a synbiotic in which the substrate is designed to be selectively utilized by the co-administered microorganism(s). A ‘complementary synbiotic’ is a synbiotic composed of a probiotic combined with a prebiotic, which is designed to target autochthonous microorganisms. Minimum criteria for the existing probiotic and prebiotic must be met for both components of a complementary synbiotic.
- Beneficial effect(s) of a synbiotic on health must be confirmed in the target host, which might include humans, companion animals, or agricultural species or a subpopulation (such as different age or developmental stage, health status, sex or living situation) thereof.
- For a synergistic synbiotic, evidence of selective utilization of the substrate must be demonstrated in the same study establishing the health benefit. The aim is to demonstrate that the combined effect is better than the estimated effects of each component separately. This step is not required for a complementary synbiotic, as it contains a prebiotic for which selective utilization has already been established.
- A synbiotic can be applied to intestinal or extra-intestinal microbial ecosystems and might be formulated into products fitting an array of regulatory categories (such as foods, non-foods, feeds, drugs or nutritional supplements).
- Implied in the definition is that safety of the synbiotic for the intended use is established.
- ‘Symbiotic’ is not a synonym of synbiotic and is incorrect in this context.

## Methods

ISAPP is a non-profit collaboration of scientists dedicated to advancing scientific excellence and providing objective, science-based information on probiotics and prebiotics. The organization’s activities are funded by companies involved in the sale of probiotics and prebiotics, but ISAPP is guided by an international, all-volunteer academic board that functions independently. This ISAPP-organized panel was composed of experts in microbiology, nutrition and gastrointestinal physiology, including many who were involved in the latest updates of the probiotic^12^ and prebiotic^13^ definitions according to ISAPP. Panellists were charged with accomplishing the following goals: consider what a synbiotic is and provide a clear, concise and testable definition; suggest the appropriate experimental conditions necessary to establish synbiotic activity; describe the evidence required to demonstrate the health benefits and establish safety; and provide guidance for stakeholders, including researchers, industry, public health professionals and regulatory agencies.

Prior to the meeting, panellists developed a discussion outline and target questions. During the meeting, panellists presented the perspectives and evidence regarding the core issues involved. Debate ensued until a consensus was achieved. After the meeting, individual panellists wrote sections of the summary, which were compiled by K.S.S., G.R.G., R.H. and M.E.S. into a draft report. This document was edited and agreed upon by all panel members. The authors would like to thank members of the ISAPP board of directors who did not directly participate in this consensus panel but who reviewed, criticized and approved this manuscript: D. Merenstein, H. Szajewska, M. Marco, E. Quigley, S. Lebeer and S. Salminen.

## An updated definition

The panel updated the definition of a synbiotic to “a mixture comprising live microorganisms and substrate(s) selectively utilized by host microorganisms that confers a health benefit on the host”. ‘Host’ microorganisms in this context include both autochthonous microorganisms (resident or colonizing the host) and allochthonous microorganisms (externally applied, such as probiotics), which, even if transiently present, do constitute a component of the host microbiota.

The panel considered defining synbiotics as simply a mixture of probiotics and prebiotics. Common to the definition of both probiotics and prebiotics is the requirement that each independently provides a health benefit and the dose of each must be adequate to independently achieve those benefit(s). However, the panel recognized the possibility that a functional synbiotic could be formulated at doses below those at which the probiotic or prebiotic could independently exert health benefits. Alternatively, a particular microorganism might lack probiotic functions even at high dosages owing to competition or other ecological effects but, in the presence of a suitable substrate, could provide a health benefit. Likewise, a novel substrate, again even at high doses, might not by itself provide benefits but could do so when combined with a selected live microorganism(s) that it can enhance. Such formulations comprise a live microorganism and a substrate that depend on the presence of one another and function in concert. Simply put, the microbial component does not necessarily have to be a standalone probiotic and the non-digestible substrate does not necessarily have to be a standalone prebiotic, but, if together they provide a health benefit, then the mixture can be called a synbiotic. This proposed definition of a synbiotic should encourage innovation in formulations by not requiring that component parts meet the strict definitions of either a probiotic or a prebiotic.

**Fig. 1:**
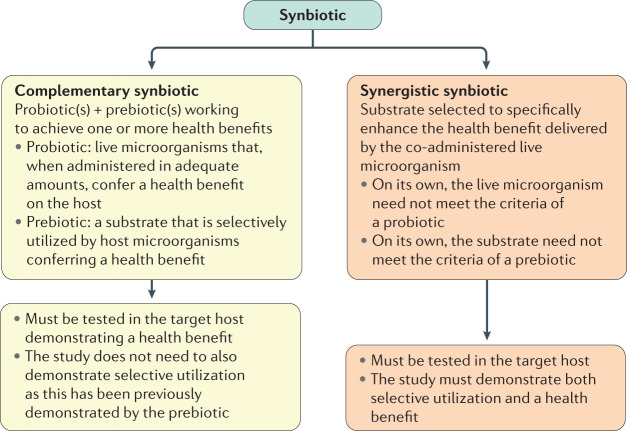
Synbiotic categories. Synbiotics can be formulated using two approaches. A complementary synbiotic comprises a probiotic plus a prebiotic (more than one of each can be used), working independently to achieve one or more health benefits. Probiotic and prebiotic components of the complementary synbiotic must meet the minimum criteria, as stipulated previously^12,13^. A synergistic synbiotic is composed of a live microorganism and a selectively utilized substrate but neither needs to meet the minimum criteria stipulated previously for probiotics and prebiotics. Instead, these components are designed to work together, with the substrate being selectively utilized by the co-administered microorganism. The panel considered whether all synbiotics should be synergistic. However, the absence of such substances today speaks to the difficulty of achieving the required evidence. The panel judged that it was more important for the definition to be useful rather than hypothetical.

However, the panel also recognized that a current common usage of the term synbiotic includes products that combine a probiotic and a prebiotic. Such a combination product might not have any evidence of co-dependent function but is instead designed for the components to work independently to promote an observed health benefit(s). The panel agreed that such a formulation could be considered a synbiotic, provided that components meet the respective probiotic^12^ and prebiotic^13^ definitions (Fig. 1). Thus, the probiotic strain(s) is chosen based on the benefits that it provides to the host, while the prebiotic is designed to promote the growth and activities of beneficial members of the indigenous microbiota and provide a health benefit^14^. Furthermore, a combination of probiotic plus prebiotic must be tested to confirm that a health benefit is conferred by the combined formulation compared with a placebo. Otherwise, the product should not be labelled a synbiotic. Based on these qualifiers, the panel retained the definition that a combination of a probiotic and a prebiotic be denoted as a complementary synbiotic (discussed later)^14^.

The other type of synbiotic previously envisioned is a synergistic synbiotic, which is a synbiotic in which the substrate is designed to be selectively utilized by the co-administered microorganisms. To be a synergistic synbiotic, the live microorganism is selected based on its ability to provide a health benefit and the substrate is chosen to primarily support the growth or activity of that selected microorganism^14^. Although the substrate might also enrich other beneficial members of the gastrointestinal microbiota, its main target is the ingested microorganism.

**Fig. 2:**
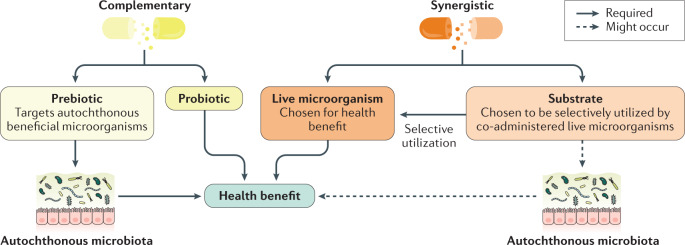
Design and mechanisms of action of complementary and synergistic synbiotics. Two approaches to designing synbiotics are represented here. The complementary approach combines a prebiotic and a probiotic that work independently to elicit one or more health benefits. The prebiotic and probiotic must each meet applicable criteria (Table 3). The prebiotic functions by modulating the resident microbiota in a manner associated with an improved health outcome. The synergistic approach selects a substrate that is utilized by the co-administered live microorganism, enhancing its functionality. Synergistic synbiotics work together (not independently) to bring about the resulting health benefits.

Designing and demonstrating the efficacy of a synergistic synbiotic is experimentally challenging. To our knowledge, nearly all synbiotics used in published clinical trials or available commercially are of the complementary form^15^, irrespective of whether they have been deliberately designed or named as such. Approaches to designing complementary and synergistic synbiotics are shown in Fig. 2.

It is important to clarify that, although synbiotics can be formulated to provide synergistic activities, the ‘syn’ prefix in the word ‘synbiotic’ is not intended to imply synergy; it means ‘together’. Of note, the term ‘symbiotic’ is often misused in this context and is not the same as synbiotic. Symbiotic, as used in biology^16^, refers to an ecological relationship in which one organism (the symbiont) lives in a long-term relationship in a natural ecosystem with another organism (the host), that is, in symbiosis.

## Characterization needed for synbiotics

A synbiotic should be characterized to the extent needed to ensure safety and a consistent performance. Live microbial component(s) of the synbiotic should have a publicly available genome sequence and annotation, be assessed for any genes of safety concern (for example, toxin production or transferrable antibiotic resistance), named using current taxonomic nomenclature and carry a traceable strain designation. The strain(s) should also be deposited into a recognized international culture collection that permits access by scientists to conduct research. In short, the safety, identity, purity and potency of the live microorganism should be clearly and accurately described according to the best available methods that meet applicable regulatory standards for the product category.

The structure and purity of the substrate should be stated and characterized by appropriate chemical analyses. This process includes testing for microbial and other contaminants as per regulatory standards for the country of sale. The level of purity required will depend on what is needed to ensure a consistent performance and safety of the product. The level of active substrate in commercial preparations of prebiotics available worldwide ranges considerably, often from 35% to 99%^17–19^. The monosaccharides and disaccharides carried over from the production process that are present in prebiotic preparations are typically digested and absorbed by the host in the upper gastrointestinal tract after oral ingestion. A relevant issue is whether the material used in the formulation is sufficient to deliver a consistent dose of the active component and result in reproducible selective utilization by microorganisms and beneficial health effect(s) in the target host. This issue highlights that studies should communicate the content of the active ingredient being tested in addition to the quantity of the overall product. For example, a 6 g dosage of 50% pure galacto-oligosaccharides would provide 3 g of the active substrate and should be reported as such.

The active ingredients of a synbiotic must be sufficiently stable. Ensuring stability of the live microbial component of a synbiotic can be challenging^20^. When live microorganisms are combined with a substrate(s) in a matrix (for example, liquid, dried or ointment), the burden is on manufacturers to ensure that the dosage of the live microorganism required to confer the stated health benefit is delivered throughout the shelf-life. Live microorganism viability is highly dependent on the matrix, storage temperature, pH and oxygen level of the product: for liquid products, the shelf-life might be as short as 1–2 weeks, for lyophilized or encapsulated products, the shelf-life might be as long as 2 years. Packaging and storage conditions must control critical factors, such as water activity and temperature, through production to distribution and usage. Furthermore, a synbiotic must undergo assessment of safety for the intended use, as described later.

## Current levels of evidence

Numerous randomized controlled trials (RCTs) in humans across a range of populations, from healthy individuals to those with acute and chronic diseases, have been conducted to examine the health benefits of putative synbiotics. Many trials have been conducted in adults with metabolic diseases, including overweight and obesity^21,22^, type 2 diabetes mellitus^23,24^ and non-alcoholic fatty liver disease^25,26^. Other outcomes, such as irritable bowel syndrome^27^, surgical infections^28,29^, chronic kidney disease^30,31^ and atopic dermatitis^32^, have also been investigated. Consequently, many systematic reviews and meta-analyses of RCTs evaluating the effect of putative synbiotics on disease targets have been published^22,23,32–35^ and this approach is an accepted means of evaluating the evidence for health benefits^36–38^. Examples are given in Table 1, which focuses on RCTs demonstrating benefits. Null trials have also been published across a variety of outcomes, including, for example, some studies aimed at the prevention of surgical infections^39,40^, treatment of obesity^41^, management of gestational diabetes mellitus^42^ and eradication of *Helicobacter pylori* infection^43,44^. However, evidence of a health benefit is not in itself sufficient to call a formulation of live microorganism(s) plus selectively utilized substrate(s) a ‘synbiotic’. Concomitant evidence of selective utilization by either the endogenous microbiota (complementary synbiotic) or the co-administered live microorganism (synergistic synbiotic) must also be generated.

**Table 1:** Human trials of orally administered combinations of live microorganisms and a substrate reporting health outcomes

Health outcome	Population studied	Synbiotic used		Refs
		Substrate component and dose	Live microorganism(s) component and dose	
Prevention of surgical infections and complications	Adults, *n* = 54	GOS (12 g per day)	*Bifidobacterium breve* strain Yakult (1×10^8^/g), *Lactobacillus casei* strain Shirota (1×10^8^/g) (3 g per day)	^96^
	Adults, *n* = 79	FOS (dose not stated, three times per day)	*Streptococcus faecalis* T-110 (60 million), *Clostridium butyricum* TO-A (4 million), *Bacillus mesentericus* TO-A (2 million), *Lactobacillus sporogenes*^a^ (100 million) (three times per day)	^97^
	Adults, *n* = 80	Inulin, β-glucan, pectin and resistant starch (2.5 g of each, twice per day)	*Pediococcus pentosaceus* 5–33:3 (10^10^), *Leuconostoc mesenteroides* 77:1 (10^10^), *Lactobacillus paracasei* subsp. *paracasei* F19 (10^10^), *Lactobacillus plantarum* 2362 (10^10^) (twice per day)	^98^
	Adults, *n* = 92	OFS (15 g, twice per day)	*Lactobacillus acidophilus* La5, *Lactobacillus bulgaricus*, *Bifidobacterium lactis* BB-12, *Streptococcus thermophilus* (4×10^9^ CFU) (three times per day)	^99^
	Adults, *n* = 46	FOS (100 mg, twice per day)	*L. acidophilus* 10 (1×10^9^ CFU), *Lactobacillus rhamnosus* HS 111 (1×10^9^ CFU), *L. casei* 10 (1×10^9^ CFU), *Bifidobacterium bifidum* (1×10^9^ CFU) (twice per day)	^45^
	Adults, *n* = 61	GOS (10 g per day)	*B. breve* strain Yakult (1×10^8^/g), *L. casei* strain Shirota (1×10^8^/g) (3 g per day)	^100^
Treatment of non-alcoholic fatty liver disease	Adults, *n* = 52	FOS (250 mg per day)	*L. casei* PXN 37, *L. rhamnosus* PXN 54, *S. thermophilus* PXN 66, *B. breve* PXN 25, *L. acidophilus* PXN 35, *Bifidobacterium longum* PXN 30, *L. bulgaricus* PXN 39 (2×10^8^ CFU per day)	^101^
	Adults, *n* = 66	FOS (dose not provided)	*B. longum* (dose not provided)	^102^
	Adults, *n* = 50	FOS (125 mg, twice per day)	*L. casei* PXN 37, *L. rhamnosus* PXN 54, *S. thermophilus* PXN 66, *B. breve* PXN 25, *L. acidophilus* PXN 35, *B. longum* PXN 30, *L. bulgaricus* PXN 39 (2×10^8^ CFU, twice per day)	^103^
	Adults, *n* = 75	Inulin HP (10 g per day)	*B. longum*, *L. acidophilus* (2×10^7^ CFU per day)	^104^
Prevention of sepsis in infants	Infants, *n* = 4,556	FOS (150 mg per day)	*L. plantarum* ATCC-202195 (~10^9^ per day)	^105^
Treatment of overweight or obesity and metabolic syndrome	Adults, *n *= 225 and *n* = 134	Litesse Ultra polydextrose (12 g per day)	*Bifidobacterium animalis* subsp. *lactis* 420 (10^10^ CFU per day)	^46,106^
	Adults, *n* = 38	FOS (250 mg, twice per day)	*L. casei* PXN 37, *L. rhamnosus* PXN 54, *S. thermophilus* PXN 66, *B. breve* PXN 25, *L. acidophilus* PXN 35, *B. longum* PXN 30, *L. bulgaricus* PXN 39 (2×10^8^ CFU, twice per day)	^107^
Treatment of T2DM and glycaemia	Adults, *n* = 62	Inulin (0.36 g, three times per day)	*L. sporogenes*^a^ (9×10^7^ CFU, three times per day)	^108^
	Adults, *n* = 81	Inulin (0.07 g/1 g bread) (120 g per day as synbiotic bread)	*L. sporogenes*^a^ (1×10^8^ CFU/g, 120 g per day as synbiotic bread)	^109^
Treatment of dyslipidaemia	Women with gestational diabetes, *n* = 70	Inulin (800 mg per day)	*L. acidophilus*, *L. casei*, *B. bifidum* (2×10^9^ CFU per day of each)	^110^
	Adults with T2DM, *n* = 78	Inulin (0.07 g/1 g bread) (120 g per day as synbiotic bread)	*L. sporogenes*^a^ (1×10^8^ CFU/g, 120 g per day as synbiotic bread)	^109^
	Adults with CHD and T2DM, *n* = 60	Inulin (800 mg per day)	*L. acidophilus*, *L. casei*, *B. bifidum* (2×10^9^ CFU/g of each per day)	^111^
	Adults with T2DM, *n* = 62	Inulin (0.36 g, three times per day)	*L. sporogenes*^a^ (9×10^7^ CFU, three times per day)	^108^
Treatment of inflammation	Elderly, *n* = 37	GOS (8 g per day)	*B. lactis* Bi-07 (10^9^ CFU per day)	^112^
	Adults, *n* = 36	FOS (1.4 g per day)	*L. acidophilus* La5, *B. animalis* ssp. *lactis* Bb-12, *Lactobacillus delbrueckii* ssp. *bulgaricus*, *S. thermophilus*, *L. paracasei* ssp*. paracasei* (2.4×10^9^ CFU per day)	^113^
	Adults with T1DM and T2DM on haemodialysis, *n* = 60	Inulin (0.8 g per day)	*L. acidophilus*, *L. casei*, *B. bifidum* (2×10^9^ CFU of each per day)	^114^
Treatment of irritable bowel syndrome	Adults, *n* = 85	FOS (100 mg, three times per day)	*Bacillus coagulans* (15×10^7^ spores, three times per day)	^115^
	Children, *n* = 71	Inulin (900 mg, twice per day)	*B. lactis* B94 (5×10^9^ CFU, twice per day)	^116^
Eradication of *Helicobacter pylori*	Adults, *n* = 76	FOS (250 mg per day)	*L. casei* PXN 37, *L. rhamnosus* PXN 54, *S. thermophilus* PXN 66, *B. breve* PXN 25, *L. acidophilus* PXN 35, *B. longum* PXN 30, *L. bulgaricus* PXN 39 (2×10^8^ CFU per day)	^117^
	Children and youth, *n* = 104	Inulin (900 mg per day)	*B. lactis* B94 (5×10^9^ CFU per day)	^118^
Treatment of polycystic ovarian syndrome	Adults, *n* = 60	Inulin (800 mg per day)	*L. acidophilus* strain T16 (IBRC-M10785), *L. casei* strain T2 (IBRC-M10783), *B. bifidum* strain T1 (IBRC-M10771) (2×10^9^ CFU/g of each per day)	^119^
	Adults, *n* = 60	Inulin (800 mg per day)	*L. acidophilus* strain T16 (IBRC-M10785), *L. casei* strain T2 (IBRC-M10783), *B. bifidum* strain T1 (IBRC-M10771) (2×10^9^ CFU/g of each per day)	^120^
Treatment of chronic kidney disease	Adults, *n* = 30	Inulin (2.2 g), tapioca-resistant starch (1.3 g) (three times per day)	*L. plantarum* (5×10^9^), *L. casei* subsp. r*hamnosus* (2×10^9^), *Lactobacillus gasseri* (2×10^9^), *Bifidobacterium infantis* (1×10^9^), *B. longum* (1×10^9^), *L. acidophilus* (1×10^9^), *Lactobacillus salivarius* (1×10^9^), *L. sporogenes*^a^ (1×10^9^), *S. thermophilus* (5×10^9^) (3 times per day)	^121^
	Adults, *n* = 66	FOS (500 mg capsule with undefined FOS dose, two capsules per day)	*L. casei*, *L. acidophilus*, *L. bulgaricus*, *L. rhamnosus*, *B. breve*, *B. longum*, *S. thermophilus* (500 mg capsule with undefined dose of microorganism, two capsules per day)	^122^
	Adults, *n* = 37	Inulin, FOS, GOS (7.5 g per day for first 3 weeks then 15 g per day for second 3 weeks)	Nine different strains across *Lactobacillus*, *Bifidobacterium* and *Streptococcus* genera (45×10^9^ CFU/day for first 3 weeks then 90×10^9^ CFU per day for second 3 weeks)	^123^
Prevention of atopic dermatitis	Pregnant women, *n* = 1,223	Newborn infants received the synbiotic: GOS (0.8 g, once per day)	Mothers received only the probiotic twice per day: *L. rhamnosus* GG (5×10^9^ CFU), *L. rhamnosus* GG LC705 (5×10^9^ CFU), *B. breve* Bb99 (2×10^8^ CFU), *Propionibacterium freudenreichii* subsp. *shermanii* JS (2×10^9^ CFU); infants received the probiotic once per day	^124^
Treatment of atopic dermatitis	Infants 12–36 months of age, *n* = 90	FOS (50 mg, twice per day)	*L. acidophilus* DDS-1, *B. lactis* UABLA-12 (5×10^9^ CFU, twice per day)	^125^
	Infants: 3 months to 6 years of age, *n* = 40	FOS (958 mg, twice per day)	*L. casei* PXN 37, *L. rhamnosus* PXN 54, *S. thermophilus* PXN 66, *B. breve* PXN 25, *L. acidophilus* PXN 35, *B. infantis* PXN 27, *L. bulgaricus* PXN 39 (1×10^9^ CFU, twice per day)	^126^
	Children: 2–14 years of age, *n* = 60	FOS (475 mg, twice per day)	*L. salivarius* PM-A0006 (2×10^9^ CFU, twice per day)	^127^

The selected studies contained in this table represent blinded, randomized, controlled trials that showed a health benefit of the combination product. Studies listed did not necessarily test both the health benefit and selective utilization by the microbiota, so we have avoided the use of the term ‘synbiotic’ in this table. Null trials and studies using the inappropriate term ‘symbiotic’ were excluded. CHD, coronary heart disease; FOS, fructo-oligosaccharides; GOS, galacto-oligosaccharides; OFS, chicory root oligofructose; T1DM, type 1 diabetes mellitus; T2DM, type 2 diabetes mellitus. ^a^*L. sporogenes* is an invalid species name; this name has been used incorrectly by some to refer to *Bacillus coagulans*.

Across studies, species from the genera *Lactobacillus*, *Bifidobacterium* and *Streptococcus* are the most commonly used live microorganisms within the formulations tested. The substrate components are usually galacto-oligosaccharides, inulin or fructo-oligosaccharides but doses vary considerably, from as low as 100 mg to as much as 10–15 g per day. For example, in a double-blind RCT, twice daily consumption of a synbiotic composed of *Lactobacillus acidophilus* 10 (10^9^ CFU), *Lactobacillus rhamnosus* HS111 (10^9^ CFU), *Lactobacillus casei* 10 (10^9^ CFU), *Bifidobacterium bifidum* (10^9^ CFU) and fructo-oligosaccharides (100 mg) 4 days before and 10 days after surgery for periampullary neoplasms resulted in a lower number of postoperative infections, a shorter duration of antibiotic therapy, fewer non-infectious complications, shorter length of hospital stay and reduced mortality than in patients receiving a placebo (sucrose)^45^. Although such low doses would not be expected to provide a prebiotic effect in complementary synbiotics, they could, in theory, be sufficient to stimulate a cognate microorganism in a synergistic synbiotic formulation. In one RCT^46^ of 225 adults with overweight and obesity, a combination of *Bifidobacterium animalis* subsp. *lactis* 420 and polydextrose (12 g per day) resulted in a relative reduction in body fat mass of 4.5%, whereas the individual treatments had no effect. However, selective utilization was not established in this study. Thus, by itself, this study does not provide sufficient evidence that the tested combination was either a complementary or a synergistic synbiotic.

In general, the appropriate dose, duration and composition of a synbiotic needed to confer a health benefit are likely to be specific to the context, including outcome and baseline host target site microbiota^47^, as well as coexisting environmental factors such as medication, habitual diet and, perhaps, yet-to-be-identified host genetic factors.

## Necessary evidence for synbiotics

A synbiotic must contain a live microorganism and a selectively utilized substrate. For complementary synbiotics, the respective components must fulfil the evidence and dose requirements for both a probiotic and prebiotic. Furthermore, the combination must be shown in an appropriately designed trial to confer a health benefit in the target host. A product containing a probiotic and a prebiotic that only has evidence for each component individually, and not as a combination product, should not be called a synbiotic. A key evidentiary requirement for a synergistic synbiotic is that there be at least one appropriately designed study of the synbiotic in the target host that demonstrates both selective utilization of the substrate and a health benefit (Box 2).

Generating this evidence requires appropriately designed, adequately powered experimental trials conducted on the target host. These studies should follow standard human trial design and reporting guidelines^48^ and consider best practices for diet–microbiota research^49^. The study should also meet the criteria outlined in CONSORT^48^ and should be registered, including a description of all outcomes, prior to recruitment. These CONSORT criteria include guiding principles for microorganisms, microbiota-related compliance and outcome measures, relevant subgroups, and statistical considerations for evaluating the microbiota as a mediator of clinical effects.

Ideally, the health benefit of a synbiotic would be superadditive, that is, it would exceed the benefit observed for the sum of the individual components. However, owing to the difficulties in demonstrating different levels of health benefit in an efficacy trial, the panel did not insist upon this aspect. Instead, we advocate requiring a measurable, confirmed health benefit of the synbiotic, which is conferred, at least in part, through selective utilization of the substrate(s) provided in the synbiotic. Although in vitro and animal models are used frequently to test the effects of probiotics, prebiotics and synbiotics^50–53^, it is our position that these have not been fully validated as predictive and that definitive tests of these interventions must be performed in the target host. Of course, model experiments can add mechanistic insights, in particular in cases for which available samples from studies in the target host have limited usefulness (for example, faeces, which are not reflective of upstream intestinal microbial or metabolic activities).

As noted, it is required that the health benefit of the synbiotic mixture be confirmed, even when established probiotic(s) and prebiotic(s) (that is, ones that meet criteria stipulated in respective definitions and are used at efficacious doses) are used to formulate components of the synbiotic. This requirement would account for any potential antagonistic effect of the combination that might diminish the health benefits of each component independently. Although unlikely, such antagonism is theoretically possible, as shown in in vitro studies whereby some carbohydrate substrates increased the production of antimicrobial compounds by probiotics^54^. Depending on which microorganism is able to utilize the substrate, which antimicrobial factors are produced, and which taxa are neutralized or killed by them, such a scenario could lead to positive or negative outcomes. Therefore, in the absence of evidence that the combination product provides a health benefit, a product should simply be labelled as “contains probiotics and prebiotics”. Sanders et al.^55^ provide a perspective on determining whether evidence from studies using one formulation (for example, a prebiotic or a probiotic alone) can be extrapolated to different formulations (for example, when combining a prebiotic and a probiotic).

Box 2 Actual and hypothetical examples to illustrate the synbiotic concept The qualifier ‘established’ refers to a probiotic or prebiotic that meets the requirements of the globally accepted definitions from International Scientific Association for Probiotics and Prebiotics consensus^12,13^. Studies are done in the target host.

**Example 1**

In a blinded four-arm study, 41 healthy volunteers received either *Bifidobacterium animalis* subsp. *lactis* Bi-07 (10^9^ CFU per day), 8 g per day xylo-oligosaccharides (XOS), the combination of both or an inert maltodextrin control for 21 days^128^. XOS enhanced bifidobacteria counts in faeces in both the synbiotic and prebiotic groups compared with the control and improved plasma lipid profiles and modulated markers of immune function in healthy adults. The lowest reported use of analgesics was observed during combination supplementation along with a reduced expression of CD19 on B cells (as markers of immune function). Thus, the combination exerted certain benefits that were not afforded by the probiotic or prebiotic alone. This study shows a prebiotic status for XOS used at 8 g per day. Previous evidence supports that *Bifidobacterium animalis* subsp. *lactis* Bi-07 at 10^9^ CFU per day met criteria for a probiotic^112,129^. Taken together, this tested product meets our definition of a complementary synbiotic. However, because an increase of *Bifidobacterium* was not observed in individuals fed the combination product, it does not meet our definition of a synergistic synbiotic.

**Example 2**

Krumbeck et al.^21^ conducted a parallel multi-arm, double-blinded randomized control trial in people with obesity (17–19 per group) to assess the effects of a synbiotic containing 5 g of galacto-oligosaccharide (GOS) and 10^9^ *Bifidobacterium adolescentis* IVS-1 on gut barrier function. The test strain had been obtained by an in vivo selection strategy intended to select for strains that would be expected to provide synergistic outcomes. In addition to the synbiotic, GOS, *B. adolescentis* IVS-1 and placebo (lactose) controls were also included. After 3 weeks of consumption, genus-specific and strain-specific quantitative real-time PCR were performed to assess changes in absolute abundances of bifidobacteria. To assess intestinal permeability, non-metabolizable sugars were measured in urine following the consumption of a sugar mixture. Although the results showed that all three treatments (GOS only, *B. adolescentis* IVS-1 only and the combination) markedly improved gut barrier function, there was no statistically significant difference among the three groups. The *B. adolescentis* IVS-1 group was significantly enriched but the addition of GOS as a synbiotic did not further increase strain abundance. Likewise, all three treatments significantly increased the absolute abundance of faecal bifidobacteria compared to baseline, but there was no statistically significant difference among the three groups. Although the combination, probiotic and prebiotic arms all improved the markers of colonic permeability and all increased faecal bifidobacteria levels, the study did not support our definition of a synergistic synbiotic.

**Example 3**

In a hypothetical study, an established prebiotic substrate (for example, GOS or inulin) at the dose shown to be both selectively utilized and have a health benefit (primary outcome showing improved probably of response) is combined with an established probiotic at the dose shown to have a health benefit in the same target host. This combination product is tested and shown to confer a health benefit compared with the control (not necessarily the same benefit as previously tested for the probiotic and prebiotic). This product would meet our definition of a complementary synbiotic.

**Example 4**

In a hypothetical study, a substrate (not an established prebiotic) at 1 g per dose is combined with 10^6^ CFU of a live microorganism (not an established probiotic). Preclinical testing suggests that the live microorganism selectively utilizes the substrate. A study tracking both health and microbiota end points in the target host comprises 10^6^ CFU per dose of live microorganism alone, 1 g per dose of substrate, 10^6^ CFU per dose of the live microorganism plus 1 g per dose of the substrate, and an inert control. Microbiota analysis supports selective utilization by the combination. Concomitantly, a health or therapeutic end point is improved by the combination. The combined effect is better than the estimated effects of each component separately. This product would meet our definition of a synergistic synbiotic.

**Example 5**

A substrate (not an established prebiotic) plus a live microorganism (not an established probiotic) is tested against the control. It is found to confer a health benefit and increase levels of bifidobacteria in faeces. This result does not provide evidence for either a synergistic or complementary synbiotic. To fulfil criteria for a synergistic synbiotic, it must be demonstrated that the health benefit and selective utilization of the substrate exceed those observed for the control and for each individual component. As the materials comprising the mixture, at the dose used, were not established probiotics or prebiotics, it does not meet our definition of a complementary synbiotic.

## Guiding principles for synbiotic research

### Design

A randomized, double-blind, placebo-controlled trial is recommended for synbiotic research. The study should be registered with accepted protocol and results systems such as ClinicalTrials.gov. Study quality factors include, but are not limited to, appropriate blinding, randomization, the allocation concealment mechanism, reporting of inclusion and/or exclusion criteria and adverse events (AEs), completion of intention-to-treat analyses, and adequate statistical power. Crossover designs might be preferred to account for the individualized nature of the gut microbiota. The wash-out period length should be based on the primary outcome with consideration for secondary outcomes; a 2-week wash-out between conditions is generally adequate for gut microbiota outcomes, although longer times might be necessary for some populations (for example, the elderly or for those with constipation or functional bowel disorders). Parallel-arm designs might be required for long-term outcomes (such as weight loss or glycaemia). The number of study groups required depends on whether the researchers intend to demonstrate synergism (Table 2). Health outcomes and selective utilization of the substrate by host microbiota must be demonstrated in the same study. When experimental trials are not feasible or ethical, observational trials, including prospective longitudinal studies, are useful; these studies must accurately capture synbiotic exposure and control for relevant confounders (such as diet or antibiotics).

**Table 2:** Evidence required for synbiotics using doses delivered in product

Composition	Dose^a^	Microbiological evidence from trial in target host	Study design	Evidence of health benefit required from trial in target host
***Complementary synbiotic***				
Prebiotic	Sufficient to result in the selective utilization by resident microbiota and a health benefit in the absence of the co-administered probiotic	No additional evidence needed beyond that for the prebiotic component	Two-arm trial of complementary synbiotic and an inert control	Complementary synbiotic is superior to control^b^
Probiotic	Sufficient to result in a health benefit in the absence of the co-administered prebiotic	No effect on resident microbiota required	Two-arm trial of complementary synbiotic and an inert control	Complementary synbiotic is superior to control^b^
***Synergistic synbiotic***				
Substrate selectively utilized by the co-administered live microorganism	Sufficient to result in the selective utilization by the co-administered microorganism	Evidence that the substrate is selectively utilized by the co-administered live microorganism	Trial of live microorganism(s), selectively utilized substrate(s), combination of microorganism(s) plus substrate(s), and control	Combined effect of synergistic synbiotic is better than the estimated effects of each component separately
Live microorganism that selectively utilizes the co-administered substrate	Sufficient to selectively utilize the co-administered substrate and result in a health benefit	Evidence that the substrate is selectively utilized by the co-administered live microorganism	Trial of live microorganism(s), selectively utilized substrate(s), combination of microorganism(s) plus substrate(s), and control	Combined effect of synergistic synbiotic is better than the estimated effects of each component separately

The unmodified term ‘synbiotic’ can be used on a commercial product label as long as the criteria for either a complementary or synergistic synbiotic are met. There is no restriction on the type of health target but it must be realistic and mechanistically driven. See Table 1 for a list of diseases and conditions targeted to date in human trials of putative synbiotics. Microbial, metabolic and health end points (or suitable biomarkers) must be tracked in the same study for a synergistic synbiotic, in the target host. It is not indicated here, but documentation of the safety of the final blended product for the intended use is required. ^a^Effective doses delivered in a commercial product must be present through the end of shelf-life. ^b^Studies documenting health benefits conferred by probiotic and prebiotic components are also required.

### Population or participants

When choosing the study population, many parameters need to be defined, including the target host (including non-human species), target life stage (for example, pregnant women, infants, children, adults or elderly) and health status (for example, healthy, at-risk, or with a diagnosed disease or condition). Furthermore, microbiome-related factors, such as recent probiotic, prebiotic, synbiotic or antibiotic use, microbiota-disrupting medications and diet can be considered as (in)eligibility criteria, depending on primary health and microbiota outcomes. A 2-week wash-in period is generally adequate for probiotics; however, certain populations might require longer wash-in periods (such as 4 weeks for those with slow transit time)^56^. The time since last antibiotic exposure will vary depending on a range of factors, including type of antibiotic, dose, duration and whether antibiotic use has occurred on multiple occasions in fairly short succession^57,58^. A general recommendation is difficult to make, although a common exclusion is individuals who have taken antibiotics 3 months prior to a study. However, at a minimum, 4 weeks since cessation of antibiotics is recommended. Non-antibiotic prescription medications should also be considered if they can influence bowel function and the microbiome^59^.

The population subgroups of interest should be specified. Clinical factors (such as sex or BMI), baseline microbiome features (such as presence of specific microbial taxa) and other microbiome-relevant factors (such as dietary fibre intake, maternal secretor status for studies on human milk oligosaccharides or birth mode) might be relevant to analyse. For studies on the elderly, consider age-associated differences in transit time, gastric acidity and diet diversity. For mother–infant dyads, consider possible vertical transmission of microorganisms from mother to infant in breast milk, microorganisms and substrates naturally present in breast milk, and the unique characteristics of immature infant microbiome.

### Intervention

The intervention should be fully described to enable replication of the study. This entails an adequate description of substrate and live microorganism dosages, specification of the microbial strain, the structure and purity of the substrate, and the timing and route of administration of the intervention. Subject compliance with an oral intervention can include measurement of the administered microorganisms in stool.

The duration of the intervention will be determined based on outcome; microbiota changes can be rapid but health outcomes will vary from days to weeks (such as constipation, stool frequency) to months (such as reduced fat mass, glycaemia). The background diet must be considered and, if possible, monitored as diet could provide a source of prebiotic substrates that are intrinsic and intact within certain foods (such as onions, wheat) as well as live microorganisms (such as fermented foods). Diet monitoring should include the use of validated methods such as the National Cancer Institute Dietary History Questionnaire (DHQ), Automated Self-Administered 24-hour dietary assessment tool (ASA24) and nutrient analysis software (such as Nutrition Data System for Research).

### Placebo or control

Design of a placebo or control is driven by the comparisons of interest. Often, a fully inert control is preferred. For formulations within pills or sachets, a low dose of highly digestible ingredients (such as maltodextrin or corn starch) or slowly fermentable fibre (such as microcrystalline cellulose) are acceptable placebos, which enables double blinding. For interventions composed of a food or beverage, the control group must be carefully considered, including differences in flavour, texture and nutrient content. These factors will also determine whether the study can be considered as a single-blinded or double-blinded design.

### Outcome

A synbiotic requires both a health outcome and a microbiota outcome in the same study. Primary and secondary health outcome(s) must be clearly specified. The microbiota outcome should be hypothesis-driven and provide insight into the microbiota-mediated mechanisms underlying the health outcome. Outcomes may include the abundance and viability of the administered live microorganism, changes in overall microbiota and/or microbiome composition, abundance of specific taxa and/or strains, microbial-derived metabolites, or others. Metabolomic analysis could also be performed using liquid chromatography–mass spectrometry, nuclear magnetic resonance or other suitable methods.

### Statistics

A statistician’s involvement in the design phase of a study will provide confidence that the sample size is sufficient for the defined outcomes using intention-to-treat analysis. Mediation analyses might be useful to assess whether microbiota effects are contributing to the health benefit. Ancillary analyses, which could include population subgroups, responders versus non-responders or exploratory microbiota analyses should be pre-specified.

Finally, as with all trials, funding sources should be transparently reported, AEs must be carefully recorded and reported, and study design, execution and reporting should comply with CONSORT guidelines^48^ to minimize study bias.

## Designing a synbiotic trial

There are multiple ways to design a synbiotic trial intended to examine health effects in the target host. Here, we provide some recommendations based on the criteria established herein to demonstrate complementary or synergistic synbiotics. Pre-specified subgroup analyses should be considered based on the relevant clinical factors (such as sex and age), baseline gut microbiota factors, dietary factors (such as typical dietary fibre intake) or other relevant factors (such as responder analysis). The number of experimental groups or ‘study arms’ required to prove efficacy and support mechanistic understanding will depend on the existing level of evidence for the component live microorganism(s) and selectively utilized substrate(s) as well as the researchers’ intent to determine whether components of the test mixture are complementary or acting as synergistic synbiotics (Table 2).

When selecting the study design and determining the duration of the intervention and (if applicable) of the wash-out period, general recommendations are difficult to make. Factors that will impact these decisions include the live microorganism dose and its ability to persist in situ as well as the characteristics of study subjects; for example, in elderly individuals or persons with slow intestinal transit time, a longer wash-out period could be required compared with a study conducted in younger individuals^56^. Parallel arm designs would be appropriate for studies that aim to demonstrate improvements in metabolic health measures, such as of adiposity or glycaemic control, as these outcomes typically require longer-term interventions (that is, 12 weeks or more). Gut microbiota changes can be rapid (within days)^60^. Currently, changes in the gut microbiota and microbial metabolites, such as short-chain fatty acids, are not accepted by regulatory bodies as measures of a health outcome^61^.

Responders and non-responders among study participants in intervention studies with probiotics and prebiotics are commonly observed, which could justify including the probability of a positive response as a study end point. In the context of synbiotics, non-responders include individuals for whom little or no change in gut microbiota composition and/or clinical end point occurs compared with placebo. Various reasons for the non-responder phenotype include limited available niches for desired gut microorganisms (either native or introduced as probiotics)^47^, limited substrates to support microbial growth^62^, host immune system expressing intolerance for specific microorganisms^63^ or a lack of specific microorganisms in the host microbiota^3^. Although many scenarios of formulations for synbiotics can be conceived, we provide both actual and hypothetical examples to illustrate the concept (Box 2).

### Trials for synergistic synbiotics

Studies on a synergistic synbiotic that compare the synbiotic to the control can provide supportive evidence but do not constitute the primary evidence needed to confirm a synergistic synbiotic. Instead, a study including the combination, the substrate alone, the live microorganisms alone and a control should be conducted. Using an appropriate statistical model, the aim is to demonstrate that the combined effect is better than the estimated effects of each component separately. Evidence of selective utilization of the substrate by the co-administered live microorganism must be obtained from the same trial demonstrating the health benefit.

### Trials for complementary synbiotics

A two-arm parallel or crossover study would be sufficient to test a complementary synbiotic. The aim is to demonstrate that the combination is better than the placebo group with a relevant health end point. As a demonstrated prebiotic is used to formulate a complementary synbiotic, we do not require that selective utilization by the indigenous microbiota be reconfirmed in this clinical trial.

### Measurement of selective utilization

Measurement of selective utilization by the microbiota might involve different approaches, including both in vitro model systems and in vivo studies in the target host. For example, selective utilization of a substrate could be demonstrated experimentally using well-established in vitro gut models^64^ that include measurement of substrates and products during a prescribed time course. Ultimately, however, studies must be done in the target host to show that specific microorganisms or taxa had been enriched or their activity enhanced by a particular substrate. For synergistic synbiotics, methods that quantify the live microorganism should be used to show that the target strain has indeed been enriched^21^ and other suitable methods can be used to show that its function has been enhanced. Microbiota features that are useful to measure can include characterization of the overall gut microbial community and microbiota diversity^65^, abundance and/or activity of specific taxa^66^, microorganism–microorganism or microorganism–host proximity^67^, the presence and/or abundance of specific microbial genes or gene clusters of interest, and/or metabolite concentrations^68^.

## Safety measures for synbiotics

Prebiotics and probiotics tested to date have a strong safety record^69–74^, and synbiotics formulated with them might also be presumed safe for the same intended uses. However, novel formulations must be suitably assessed for safety. Unfortunately, historically, many probiotic and prebiotic intervention trials have not adequately reported the types and frequency of AEs or serious AEs, perhaps owing to expectations that, as food ingredients, these products were inherently safe or that AEs could be due to failure to comply with norms for reporting harms in RCTs. Nonetheless, clear guidance for reporting AEs and serious AEs is provided by CONSORT^75^ and these standards should be followed. Describing such events as “unrelated to the study product”, without justification for this statement, is unacceptable.

A systematic review of 384 interventions involving prebiotics, probiotics and synbiotics found that no safety-related data were reported in 37% of the RCTs and 89 studies only used generic statements to describe AEs^76^. Up to 98% of studies did not provide a definition of AEs or serious AEs, the number of participant withdrawals due to AEs, or the number of AEs and serious AEs per study group. Taken together, these results are evidence that some studies inadequately collect or report data on AEs. van den Nieuwboer and colleagues^72,77–79^ reviewed the AEs reported in studies with probiotic and synbiotic interventions and classified them according to the Common Terminology Criteria for Adverse Events system^80^; they found that the incidence of AEs in each category was either no different from or often lower in the control groups than in the treatment group.

Thorough safety assessment of a synergistic synbiotic requires consideration that the added microorganism will express enhanced functionality in the presence of a targeted substrate such as improved growth or altered metabolic or physiological activity in vivo. This aspect suggests that safety assessments conducted on the live microorganism in isolation might not be sufficient to enable a conclusion about its safety when paired with a substrate that alters physiology or might in effect alter the dose delivered in vivo. Implications of such an interaction should be considered when assessing the safety of synergistic synbiotics.

## Implications of this consensus

The panel recognizes the importance of developing a scientifically valid definition of the term synbiotic. The intention is to provide clarity for a diverse range of stakeholders, including consumers, regulators, health-care providers, researchers, industry and scientific organizations communicating about synbiotics. Table 3 is designed to clearly lay out the criteria for synbiotics in comparison to probiotics and prebiotics. Within the commercial supply chain, many different industry sectors, including ingredient suppliers, end-product manufacturers and retailers are very interested in how synbiotics are defined. As synbiotic products are being used more frequently in clinical trials, it is incumbent upon scientists to clarify the appropriate use of this term as has been done for the terms ‘probiotic’^12^ and ‘prebiotic’^13^.

**Table 3:** Minimum criteria to appropriately use the terms ‘probiotic’, ‘prebiotic’ and ‘synbiotic’

Substance	Safe for intended use	Identity characterized	Scientifically valid name	Strain designated	Microorganism deposited in international culture collection	Mechanism of action linked to microbiota	Selective utilization of substrate		Study in target host demonstrating both:		Proper conditions of use
							By resident microbiota	By co-administered live microorganism	Health benefit	Selective utilization of substrate	
Probiotic	☑	☑	☑	☑	☑	NA	NA	NA	☑	NA	☑
Prebiotic	☑	☑	☑	NA	NA	☑	☑	NA	☑	☑	☑
Synbiotic											
Complementary synbiotic	☑	☑	☑	☑	☑	☑	☑	–^a^	☑	NR	☑
Synergistic synbiotic	☑	☑	☑	☑	☑	☑	–^a^	☑	☑	☑	☑

Probiotics are live microorganisms that, when administered in adequate amounts, confer a health benefit on the host. A prebiotic is a substrate that is selectively utilized by host microorganisms conferring a health benefit. Synbiotics are a mixture comprising live microorganisms and substrate(s) selectively utilized by host microorganisms that confers a health benefit on the host. A complementary synbiotic is a mixture of a probiotic plus a prebiotic. A synergistic synbiotic is a synbiotic in which the substrate is designed to be selectively utilized by the co-administered microorganisms. A synbiotic must meet the evidence required for either complementary or synergistic synbiotics. All substances should be made available to the scientific community for validation of research findings. ^a^The intent of a synergistic synbiotic is for the substrate to support the growth and/or activity of the co-administered live microorganisms but selective utilization by the resident microbiota is not a disqualifier. The prebiotic component of a complementary synbiotic must be selectively utilized by the resident microbiota, but if it is also utilized by the co-administered probiotic, it is not a disqualifier. NA, not applicable; NR, not required.

Although the term ‘synbiotic’ might not be as well recognized as probiotics and prebiotics, it is nonetheless found on product labels, in popular press articles and in the scientific literature. The first mention of a synbiotic was in 1995 yet, according to PubMed searches, in 2019, 269 papers were published using the term. Consumer exposure is expected to increase. Thus, it is hoped that the definition herein is clear and widely accepted and will counter misuse of the term, including by scientists^8^. Table 3 provides the minimum criteria for the correct use of terms associated with synbiotics.

### The importance of context

The panel urges stakeholders to carefully consider context for communications to consumers on the health benefits of synbiotics. The overall impression of any communication should lead consumers to understand the claim in a manner that is consistent with the evidence; any misleading use of the term constitutes misuse. Communication of the tested health benefit must accurately reflect what was reported in the clinical trial; results should not be extrapolated to health conditions, populations or synbiotics that have not been studied.

### Regulators

Regulatory authorities are primarily focused on two issues: product safety and product labelling. These encompass both truthfulness and compliance with regulatory statutes. Even if the term synbiotic is not included in governmental guidelines or regulations, the use of our proposed scientific definition of the term will aid regulatory oversight of products labelled as ‘synbiotic’.

Regulatory statutes will differ with regard to geographical regions, regulatory categories, types of allowable claims and premarket approval. Furthermore, different standards exist for manufacturing, efficacy and safety depending on geographical region and product category. The term synbiotic does not stipulate a regulatory category, so (simply stated), regulatory requirements for a synbiotic would need to meet those that apply to the category (for example, drug, food or supplement) of the marketed product.

Regulatory complications can result in regions that impose probiotic-specific regulations. For example, Canada^81,82^, Italy^83^, Argentina^84^, Chile^85^, Colombia^86^ and Brazil^87^ have requirements specific to probiotic foods or supplements. Furthermore, there is a proposal^88^ under consideration by Codex Alimentarius that could result in probiotic-specific global standards^89^. The Codex Alimentarius is a collection of standards, guidelines and codes of practice adopted under the auspices of the Food and Agricultural Organization of the United Nations and World Health Organization to protect consumer health and promote fair practices in food trade. Codex Alimentarius standards could affect probiotic products traded globally. Could product manufacturers potentially avoid these regulations by labelling a product as a synbiotic? Without the use of the term probiotic or prebiotic, relevant specific regulations would not be triggered. Perhaps this approach would not provide a marketing advantage, as consumer recognition and demand for synbiotic products is currently not as advanced as for probiotics and prebiotics. However, regions supporting probiotic-specific (or prebiotic-specific) regulations will need to address how the concept of synbiotics fits into those regulations. Our stipulation is that manufacturers of complementary synbiotics should meet current probiotic or prebiotic regulations.

One regulatory consequence of probiotic and prebiotic definitions is that the European Union has determined that labelling a food product as such amounts to an implied health claim^90^. As health claims in the European Union must be approved, the use of ‘probiotic’ or ‘prebiotic’ on a food label is subject to a health claim approval process. Despite controversy surrounding this situation, we can expect that the European Union might adopt a similar position for the use of the term ‘synbiotic’ because it also requires evidence of a health benefit.

### Scientists

Synbiotics present challenges for researchers, including deciphering mechanisms underlying the health benefit and proving that the microbiota are modulated via complementary or synergistic means. Unless appropriate experimental methods of verification are used, the product under assessment should not be referred to as ‘synbiotic’.

As for probiotics, prebiotics and synbiotics, it might be possible a priori to stratify study participants to enhance either the magnitude of the response or the number of responders to a given synbiotic treatment. The baseline composition of gut microbiota of study participants could be one useful stratification factor^91,92^. This stratification might improve mechanistic understanding of observed effects and could be useful for characterizing responders and non-responders. Ultimately, this approach could enable better translation of clinical trial outcomes to those who are likely to benefit.

Scientists involved in the publication process (authors, editors, reviewers) should use the term synbiotic correctly and reject erroneous or unsupported use of the term. Even if a given treatment fails to demonstrate an effect (that is, acceptance of the null hypothesis) in a well-conducted study, such results still warrant publication^93^.

### Industry

The business community plays a vital part in translating the outcomes of fundamental and clinical research into commercial products that can benefit consumers. To the extent that industry funds research, it is essential that studies adhere to well-established guidelines to manage conflicts of interest and minimize bias^94,95^. Further, industry bears the duty of responsible manufacture, quality control and marketing of synbiotic products. It is incumbent on industry to follow good manufacturing practices, engage in truthful labelling and product promotion as well as adhere to appropriate use of the term ‘synbiotics’.

### Media

The media (TV, radio, newspapers, magazines, websites and social media) have an increasingly central role in communicating science. These efforts require commitment and skill to craft clear, simple messages that are true to the science. Too often, scientists or their institutions do not effectively translate complex research findings so that they can be understood by the lay public. At times, overextended or inflated representations of research findings can be communicated through official academic or journal press releases. Communicators are therefore encouraged to distinguish between association studies and those presenting causal evidence, to describe single studies in the context of the totality of evidence, to avoid overgeneralizing results (for example, from model organisms such as mice) and to note study limitations. This requirement is true for null studies as well as those suggesting benefits from the interventions being tested. In this context, the media should adopt the scientific definition of synbiotics herein.

## Conclusions

This Consensus Statement provides a new definition for a ‘synbiotic’ based on review by a panel of experts. To summarize (Box 1), a synbiotic is a mixture comprising live microorganisms and substrate(s) selectively utilized by host microorganisms that confers a health benefit on the host. Two categories of synbiotics are recognized. A complementary synbiotic is composed of a probiotic and a prebiotic that together confer one or more health benefits but do not require co-dependent function; the components must be used at doses that have been shown to be effective for the components alone. A synergistic synbiotic contains a substrate that is selectively utilized by the co-administered live microorganism(s). Synbiotic products are not confined to human applications but could also include companion animals and livestock. They might also be directed to specific subpopulations (age, sex, health status) of the target species. The definition can also be applied to intestinal or extra-intestinal microbial ecosystems. The hope is that, going forward, this updated definition will be utilized and that it will aid in advancing synbiotic research, improve stakeholder understanding and enable better communication to consumers.
